# The Contribution of Iron to Protein Aggregation Disorders in the Central Nervous System

**DOI:** 10.3389/fnins.2019.00015

**Published:** 2019-01-22

**Authors:** Karina Joppe, Anna-Elisa Roser, Fabian Maass, Paul Lingor

**Affiliations:** ^1^Department of Neurology, University Medical Center Göttingen, Göttingen, Germany; ^2^Center for Biostructural Imaging of Neurodegeneration, Göttingen, Germany; ^3^German Center for Neurodegenerative Diseases, Göttingen, Germany; ^4^Rechts der Isar Hospital, Technical University of Munich, Munich, Germany

**Keywords:** iron, protein aggregation, neurodegeneration, disease mechanism, iron chelator

## Abstract

The homeostasis of iron is of fundamental importance in the central nervous system (CNS) to ensure biological processes such as oxygen transport, mitochondrial respiration or myelin synthesis. Dyshomeostasis and accumulation of iron can be observed during aging and both are shared characteristics of several neurodegenerative diseases. Iron-mediated generation of reactive oxygen species (ROS) may lead to protein aggregation and cellular toxicity. The process of misfolding and aggregation of neuronal proteins such as α-synuclein, Tau, amyloid beta (Aβ), TDP-43 or SOD1 is a common hallmark of many neurodegenerative disorders and iron has been shown to facilitate protein aggregation. Thus, both, iron and aggregating proteins are proposed to amplify their detrimental effects in the disease state. In this review, we give an overview on effects of iron on aggregation of different proteins involved in neurodegeneration. Furthermore, we discuss the proposed mechanisms of iron-mediated toxicity and protein aggregation emphasizing the red-ox chemistry and protein-binding properties of iron. Finally, we address current therapeutic approaches harnessing iron chelation as a disease-modifying intervention in neurodegenerative disorders, such as Parkinson’s disease, Alzheimer’s disease, and amyotrophic lateral sclerosis.

## Introduction

Neurodegenerative disorders (NDDs) rapidly gain importance due to their age-related prevalence and the resulting socio-economic burden (Hindle, 2010; Abbott, 2011). Aging is one of the main risk factors for NDDs (Ashraf et al., 2018) and the constantly growing life expectancy will result in their increased prevalence (Oeppen and Vaupel, 2002). Although different NDDs present a variety of symptoms ranging from cognitive, motor, sensory and/or autonomic failure, neuronal loss is the shared characteristic. Therefore, it is of great relevance to identify common pathophysiological features that are present in multiple NDDs to elucidate general mechanisms of neurodegeneration and potential pathways for intervention.

Iron does not only play a main role in cellular senescence but also in NDDs such as Parkinson’s disease (PD), Alzheimer’s disease (AD), amyotrophic lateral sclerosis (ALS) or prion diseases (PrD) (Ashraf et al., 2018). As protein aggregation is another shared hallmark among many NDDs (Iadanza et al., 2018), it is suggestive to assume a mutual interplay of iron and protein aggregation amplifying their detrimental effects. In addition to iron, other transition metals such as copper and manganese are also considered in the pathogenesis of NDDs. This review, however, is focusing on the role of iron in protein aggregation disorders and the reader is referred to alternative publications on the role of other metals (e.g., Carboni and Lingor, 2015; Kim et al., 2018).

In this review, we discuss the mode of action of iron in NDDs and proposed mechanisms of iron-mediated protein aggregation. Finally, we outline recent therapeutic approaches targeting iron as promising treatment option for NDDs.

## Iron Dyshomeostasis and Accumulation

Iron is the most abundant trace metal in the human brain, present in neuronal and glial cells. It acts as a catalytic center for multiple enzymes and supports the synthesis of DNA, neurotransmitters and myelin in the brain. Furthermore, it participates in oxygen transport, neurotransmitter metabolism and mitochondrial respiration (Ward R.J. et al., 2014; Ashraf et al., 2018).

Iron metabolism is tightly regulated by iron responsive elements (IREs). In the presence of iron, binding of the iron regulatory protein (IRP) to IREs controls the translation of mRNAs related to proteins of iron metabolism, e.g., iron import, export and storage proteins. Under physiological conditions, extracellular ferric iron (Fe^3+^) is mainly bound to the glycoprotein transferrin (Tf), being delivered to cells by transferrin receptors (TfR). Non-transferrin-bound ferrous iron (Fe^2+^) is mainly transported into the cell via the divalent metal-ion transporter 1 (DMT1) (Hider and Kong, 2013; Jiang et al., 2017). DMT1 is also an important player in mitochondrial uptake of Fe^2+^ (Wolff et al., 2018). Intracellularly, ferritin is the major iron storage protein complex. Before storage in both H- or L-ferritins, Fe^2+^ is oxidized by H-ferritin to Fe^3+^. Under normal conditions, the labile Fe^2+^ pool and ferritin molecules are at an equilibrium. The export of Fe^2+^ is regulated by ferroportin-1 (FPN1) being controlled by hepcidin (Hider and Kong, 2013; Ashraf et al., 2018).

Impaired iron metabolism coupled with its accumulation in various brain regions are hallmarks of physiological aging. H-and L-ferritins also are more abundant with age (Zecca et al., 2001). During life, both ferritin subunits increase in their concentration within the SN but stay constant within the locus coeruleus. Both regions are important target areas for PD whereas the locus coeruleus is also affected in AD (Zecca et al., 2004a). Brains of patients suffering from NDDs, e.g., PD and AD, are lacking the age-associated rise of both ferritins. In PD, reduced ferritin levels in SN and pathological iron accumulation were found (Dexter et al., 1991; Connor et al., 1995).

Excessive ROS production resulting in oxidative stress is a common feature of NDDs and accumulated redox-active iron triggers ROS formation by the *Fenton* and *Haber-Weiss* reactions, providing the basis for catalyzed oxidation processes. Accordingly, iron reacts with hydrogen peroxide, which is a by-product of the mitochondrial respiration and intracellularly abundant, resulting in hydroxyl free radicals (HO•). Therefore, iron fosters the formation of ROS that lead to oxidative stress, inducing mitochondrial dysfunction and cell death (Zecca et al., 2004b).

This said, the reasons for iron accumulation and its precise effects on pathomechanisms in neurodegeneration remain still incompletely understood. Its contribution to the aggregation of disease-relevant proteins may be a major effector of its toxicity in NDDs.

## Protein Aggregation

A shared hallmark of numerous NDDs is protein aggregation. For example, α-synuclein aggregates are the main components of Lewy bodies in PD (Spillantini et al., 1998), whereas neurofibrillary tangles and plaques in AD are composed of Tau and Amyloid beta (Aβ), respectively (Glenner and Wong, 1984; Brion, 1998). Aggregation of TDP-43 or SOD1 are observed in ALS (Brown, 1998; Neumann et al., 2006). Recent data demonstrate, however, that aggregation of one particular protein is not specific for one disease (e.g., Cisbani et al., 2017; Trist et al., 2018). Under physiological conditions, the ubiquitin proteasome system (UPS), autophagosomes and chaperone activity ensure the clearance of protein aggregates (Stroo et al., 2017). However, genetic or environmental factors can disturb the balance of aggregate formation and clearance, so that native soluble proteins or peptides start misfolding and assemble into insoluble beta-sheet oligomers and protofibrils. This filamentous aggregation results in amyloid fibrils and protein inclusion formation. For different disease-dependent proteins this aggregation process is likely to follow similar pathways (Soto and Pritzkow, 2018).

Whereas for protein inclusions a possible neuroprotective role is still discussed, oligomers and protofibrils of the above-mentioned species are very likely neurotoxic. Amyloid structures are believed to impair axonal transport, DNA transcription and the UPS, and trigger mitochondrial dysfunction, synaptic dysfunction and oxidative stress (Dhouafli et al., 2018; Iadanza et al., 2018). Furthermore, oligomers increase the lipid bilayer conductance and, therefore, induce calcium dyshomeostasis (Verma et al., 2015). Altogether, these mechanisms contribute to cellular dysfunction and cytotoxicity.

## Iron and Protein Aggregation

**FIGURE 1:**
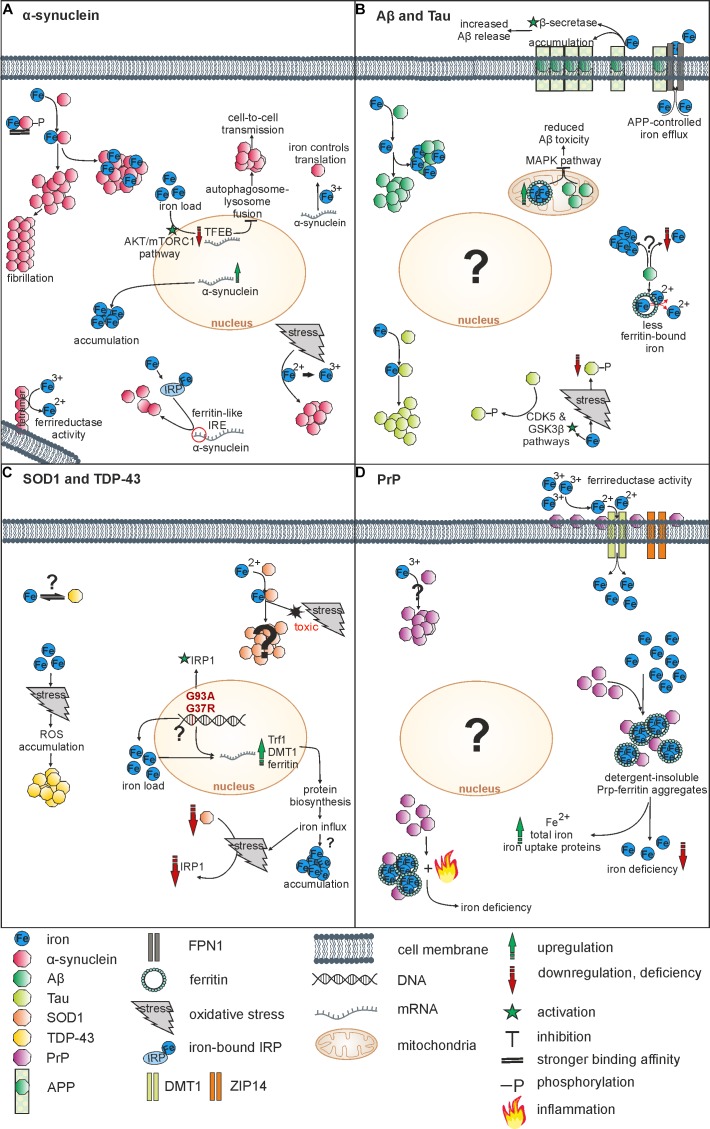
Overview on relevant interactions of iron and NDDs-associated proteins. **(A)** Iron induces α-synuclein aggregation by direct binding or via oxidation. Indirectly, iron also influences α-synuclein on its transcriptional and translational level. α-synuclein acts as a ferrireductase and can induce iron accumulation by overexpression. **(B)** Iron fosters aggregation of both Aβ und Tau by binding. Whereas Aβ reduces levels of ferritin-bound iron, an overexpression of mitochondrial ferritin reduces Aβ toxicity. APP controls iron efflux and together with iron it affects the Aβ release. Furthermore, there is evidence for both, Aβ-induced iron accumulation and Aβ-induced iron depletion. Whereas iron increases Tau-phosphorylation via CDK5 and GSK3ß pathways, iron-induced oxidative stress reduces Tau-phosphorylation. **(C)** Iron binds SOD1, inducing oxidative stress and toxicity. Mutations of SOD1 lead to an upregulation of iron metabolism proteins followed by iron influx. Iron is suggested to affect TDP-43 aggregation indirectly via oxidative stress-mediated ROS accumulation. An interaction of iron and TDP-43 has not been objectified so far. **(D)** PrP operates as a ferrireductase partner of ZIP14 and DMT1 increasing Fe^3+^ uptake. Furthermore, PrP-ferritin aggregates induce iron deficiency and an upregulation of total iron, Fe^2+^ and iron uptake proteins. Inflammation processes may contribute to iron deficiency. Vice versa, Fe^3+^ triggers PrP accumulation within the cell.

Via interaction with redox-active metal ions, amyloidogenic forms of, e.g., Aβ or α-synuclein triggered ROS production and oxidative cytotoxicity (Liu et al., 2011; Deas et al., 2016). Especially iron was shown to enhance aggregation processes of α-synuclein (Ostrerova-Golts et al., 2000), Aβ (Rottkamp et al., 2001) or Tau (Sayre et al., 2000). How iron enhances protein aggregation is not fully understood, but two distinct mechanisms are considered as relevant. First, the direct binding of iron to amyloidogenic proteins, and second, an indirect iron-mediated process, where the above-mentioned *Fenton* and *Haber-Weiss* reaction of Fe^2+^ triggers aggregation by ROS production and resulting oxidative stress. An overview on relevant interactions of iron and below-mentioned proteins is shown in Figure 1.

### α-Synuclein

α-Synuclein is a 140 amino acid protein expressed in neuronal cytosol and presynaptic terminals that is thought to participate in vesicle packaging, release and trafficking as well as in membrane remodeling. Furthermore, interactions of α-synuclein with histones as well as with nuclear DNA are suspected, but its concrete function in the nucleus and presynaptic terminals needs to be further investigated (Bendor et al., 2013; Rocha et al., 2018). α-Synuclein is intensively studied in regard to the pathophysiology of PD, since some inherited forms of PD can result from point mutations and from overproduction of α-synuclein through multiplications of the SNCA gene encoding the human α-synuclein protein. In total, six point mutations (A30P, E46K, H50Q, G51D, A53T, and A53E) of the SNCA gene were identified so far leading to a biophysical change of amino acid substitutions (Uchihara and Giasson, 2016). Therefore, models overexpressing α-synuclein or a mutated form are used to understand the role of α-synuclein and its interaction with iron (e.g., Zhu et al., 2016; Carboni et al., 2017).

Analyzing the interaction of iron and α-synuclein, different studies could show that α-synuclein fibrillation can be induced by iron (Uversky et al., 2001; Golts et al., 2002; Kostka et al., 2008). *In vitro*, using BE-M17 neuroblastoma cells iron had stronger effects on aggregation of A53T and A30P mutant compared to wild-type α-synuclein (Ostrerova-Golts et al., 2000). *In vivo*, iron treatment showed reduced survival of α-synuclein mutant (A53T, A30P) and α-synuclein wild-type flies compared to w1118 wild-type controls, but only the mutant flies showed a strong motor decline (Zhu et al., 2016). Transmission electron microscopy resolved that *ex vivo* Fe^3+^ addition to wild-type and mutant (A53T, A30P, E46K) α-synuclein generates fibrils, resembling fibril conformations formed without iron incubation, whereas copper addition to mutant α-synuclein led to the formation of amorphous aggregates. These results indicate that the fibril morphology is metal-specific (Bharathi et al., 2007).

Even micromolar concentrations of Fe^3+^ increased α-synuclein aggregation and produced larger SDS-resistant oligomers. Since H_2_O_2_ treatment did not lead to the same effects as iron treatment, oxidation of α-synuclein *per se* cannot be the reason for the oligomerization, showing that trivalent ions play a relevant role (Kostka et al., 2008). However, further *in vitro* studies showed that Fe^2+^ also promoted α-synuclein aggregation, transmission and affected viability of SK-N-SH and SN4741 cells (Li et al., 2011; Xiao et al., 2018). Specifically, in SK-N-SH cells α-synuclein aggregation was increased around the nucleus (Li et al., 2011). Under aerobic conditions *in vitro*, Fe^2+^ treatment caused a polymerization into antiparallel soluble α-synuclein oligomers, whereas under anaerobic conditions both Fe^2+^ and Fe^3+^ induced parallel ß-sheet aggregates (Abeyawardhane et al., 2018).

How iron influences α-synuclein aggregation is not completely understood. However, α-synuclein has a high metal binding affinity and it is known that both Fe^2+^ and Fe^3+^ can bind α-synuclein, revealing a binding constant of 1.2 x 10^13^ M^−1^ for Fe^3+^ and 5.8 x 10^3^ M^−1^ for Fe^2+^ (Peng et al., 2010). Fe^2+^ binds at the C-terminus, specifically at Asp-121, Asn-122, and Glu-123 (Binolfi et al., 2006) and the binding affinity is increased in phosphorylated (pY125 or pS129) α-synuclein (Lu et al., 2011). Additionally, Fe^3+^ can bind α-synuclein, having two binding sites, which are likely at the C-terminus (Davies et al., 2011).

Interestingly, there is a close homology in the 5′-UTR of human α-synuclein mRNA to the IRE of the ferritin mRNA, which could explain the regulation of α-synuclein levels by intracellular iron (Friedlich et al., 2007). Fe^3+^ was suggested to control the translation of α-synuclein mRNA since the iron chelator deferoxamine (DFO) highly decreased the polysome-associated endogenous α-synuclein mRNA in HEK293 cells (Febbraro et al., 2012). Furthermore, a knockdown of the IRP in SK-N-SH cells enhanced α-synuclein aggregation by upregulation of α-synuclein transcription, indicating that iron partially controls the aggregation process through the IRE/IRP system (Li et al., 2011). In addition to its direct binding there is thus evidence that iron also influences α-synuclein on a transcriptional and translational level.

Iron also promotes α-synuclein aggregation indirectly by regulating the nuclear transcription factor EB (TFEB), which is a transcriptional regulator of the autophagosome-lysosome pathway. Iron enrichment decreased the TFEB expression and inhibited its nuclear translocation through the activation of the Akt/mTORC1 pathway resulting in increased α-synuclein aggregation in cell lysates by the inhibition of TFEB-mediated autophagosome-lysosome fusion. Furthermore, iron increased α-synuclein cell-to-cell transmission, which was attenuated by TFEB overexpression (Xiao et al., 2018). As an indirect pathway, oxidative stress contributes to the iron-induced aggregation. This was further supported by the finding that supplementation of the antioxidative vitamin E attenuated aggregation in SK-N-SH cells (Li et al., 2011). Additionally, an *ex vivo* study indicated that oxidative stress enhances α-synuclein aggregation indirectly via oxidation of iron from Fe^2+^ to Fe^3+^ (Levin et al., 2011).

Vice versa, iron levels were increased by the overexpression of α-synuclein itself in primary midbrain neurons and PC12 cells which were analyzed with particle induced X-ray emission. Iron accumulation quantified by X-ray fluorescence was specifically observed within the perinuclear regions of PC12 cells (Ortega et al., 2016).

Furthermore, α-synuclein was also found to act as a ferrireductase, reducing Fe^3+^ to Fe^2+^ (Davies et al., 2011). The ferrireductase active form of α-synuclein is suggested to be a membrane-associated helical-rich tetramer (McDowall et al., 2017; Angelova and Brown, 2018). Previous studies suggest a resistance of the tetramer to fibril and aggregate formation, making the tetramer to an interesting subspecies *in vivo* (Bartels et al., 2011; Wang W. et al., 2011; Dettmer et al., 2015).

Regarding treatment strategies to reduce or prevent α-synuclein aggregation, iron seems to be a promising target. Iron recycling by Nramp1 (Soe-Lin et al., 2009) was shown to degrade microglial α-synuclein oligomers *in vitro* and *in vivo*, highlighting natural defense mechanisms in iron overload conditions (Wu et al., 2017). Furthermore, different iron chelators showed various beneficial effects in preclinical cell or animal PD models (e.g., Sangchot et al., 2002; Mandel et al., 2004; Billings et al., 2016; Finkelstein et al., 2016, 2017; Carboni et al., 2017; Das et al., 2017).

Iron and α-synuclein thus influence each other mutually: whereas iron contributes to α-synuclein aggregation by direct binding and indirectly via oxidative stress and transcription factors, α-synuclein shows ferrireductase activity influencing iron homeostasis.

### Aβ and Tau

Aβ is a metalloprotein consisting of 39–43 residues that is derived from the transmembrane amyloid precursor protein (APP) by proteolytic cleavage. Aβ aggregates are found as amyloid plaques in AD and Aβ plays a role in metal sequestration and homeostasis, synaptic activity and neuronal plasticity (Smith et al., 2007; Rajasekhar et al., 2015). Aβ was found to be strongly colocalized with brain iron in risk patients for AD measured by magnetic resonance imaging and positron emission tomography (Van Bergen et al., 2016). X-ray studies analyzing Aβ plaques in cortex tissue of transgenic mice and Aβ plaque cores of AD patients confirmed a direct correlation of iron and Aβ localization, suggesting the formation of an iron-Aβ complex (Telling et al., 2017; Everett et al., 2018). *Ex vivo*, Fe^3+^ was found to promote aggregation of Aβ1-40 and Aβ1-42 visualized by fluorescence spectroscopy and atomic force microscopy (Tahmasebinia and Emadi, 2017). Fe^3+^ was also shown to bind Aβ using the phenolic oxygen of tyrosine 10 as binding site (Miura et al., 2001). Fe^3+^-mediated generation of Aβ aggregates was also reported *in vitro*. However, these aggregates were shorter and less ordered than in iron-free Aβ incubation (Liu et al., 2011). In drosophila, the affinity of Aβ for iron is mediated by three N-terminal histidines enhancing Aβ dimerization and leading to histidine-dependent oxidative damage (Ott et al., 2015).

Furthermore, treatment with iron chelators (clioquinol, YM-F24) increased survival and locomotor function of flies expressing Aβ1-42 compared to control wild-type flies, highlighting the relevance of oxidative stress for neurotoxicity of Aβ (Rival et al., 2009; Liu et al., 2011). Iron chelation also showed beneficial effects in mammalian models (Fine et al., 2015; Zhao et al., 2017) and reduced Aβ1-42 aggregation in an *ex vivo* study (Tahmasebinia and Emadi, 2017). Other studies showed that the presence of the important iron storage protein ferritin has beneficial effects on Aβ pathology. Accordingly, *in vitro* and *in vivo*, mitochondrial ferritin reduced neurotoxic effects exerted by Aβ (Wu et al., 2013; Wang P. et al., 2017). The overexpression of mitochondrial ferritin in SHSY5Y cells prevented the activation of the MAPK signaling pathway, which is related to oxidative stress-induced cell death (Wu et al., 2013).

Iron also strongly influences APP. Treating SHSY5Y cells with Fe^3+^ caused APP accumulation in membrane-enriched cellular fraction and increased activity of β-secretase that both triggered increased release of Aβ1-42 (Banerjee et al., 2014). Another study using SHSY5Y cells confirmed increased APP steady state levels and APP production after iron treatment (Rogers et al., 2016).

Furthermore, APP influences iron export by controlling the persistence of FPN1 on the neuronal surface, even if it does not function as a ferroxidase (Wong et al., 2014). There is evidence that Aβ induces iron accumulation in a cell-free *ex vivo* study (Everett et al., 2014b). Other studies suggest that Aβ controls the redox-activity of iron and reduces iron chemically *in vivo* (Everett et al., 2018). Accordingly, Aβ was able to inhibit ascorbate-dependent hydroxyl radical generation by free Fe^3+^ (Nakamura et al., 2007). Furthermore, interaction with Aβ led to a reduction of ferrihydrite (ferritin-bound iron) to pure redox-active Fe^2+^ (Everett et al., 2014a).

Iron was not only shown to modulate the aggregation of Aβ but also of Tau (Kim et al., 2018). Tau is a microtubule-associated protein that is the main component of neurofibrillary tangles in AD. Mostly, it is located in axons and sometimes in dendrites (Nisbet et al., 2015). So far, little is known about the interplay of iron and Tau and present studies are not consistent. Iron treatment enhanced Tau aggregation in iron-enriched hippocampal regions and iron was shown to bind Tau (Sayre et al., 2000). Some studies showed that only trivalent metal ions, as Fe^3+^, trigger Tau aggregation but not divalent ions (Yamamoto et al., 2002; Bader et al., 2011). Fe^3+^-generated Tau oligomers were even more stable than DMSO-generated ones (Nübling et al., 2012). However, a recent electrochemical study showed that both, Fe^3+^ and Fe^2+^, bind Tau at different binding sites, inducing a structural change, which was more pronounced with Fe^2+^ (Ahmadi et al., 2017).

Iron-induced oxidative stress reduced Tau phosphorylation by interfering with the function of the CDK5/p25 system of hippocampal neurons (Egaña et al., 2003). Another study using primary cultures of rat cortical neurons showed iron-induced Tau phosphorylation by activation of GSK3 (Lovell et al., 2004). Ebselen, an organo-selenium compound with antioxidant activity, inhibits the CDK5 and GSK3β pathway leading to less Tau phosphorylation. These effects were not only caused by the antioxidant effects of Ebselen, but rather by the inhibition of DMT1 in SHSY5Y cells (Xie et al., 2012). The same effects on CDK5 and GSK3β as well as on Tau phosphorylation were shown with the iron chelator DFO analyzing APP/PS1 transgenic mouse brains (Guo et al., 2013).

In summary, iron binds both, Aβ and Tau, partially modifying their structure and fostering their aggregation process. Furthermore, the Aβ and Tau phenotypes can be partially rescued by iron chelation.

### SOD1 and TDP-43

SOD1 is an abundant antioxidant protein predominantly located within the cytosol. Its aggregation leads to, e.g., neuronal degeneration and changes in DNA/RNA metabolism, neurofilament and axonal transport (Pasinelli and Brown, 2006). An NMR study showed binding of iron to SOD1 with binding sites closely located to Cu/Zn binding pockets leading to Fe^2+^-bound SOD1 complexes, which are likely toxic (Lim and Song, 2015). Even if there is no direct evidence for iron-induced SOD1 aggregation, several studies emphasize a coherence of iron load and SOD1 pathology.

Therapeutic approaches showed that in SOD1 mouse models (G93A, G37R, G86R) iron chelators [VK-28, M30, HLA20, VAR-ced, deferiprone (DFP)] extended lifespan, increased locomotor function and motoneuron survival and decreased oxygen free radicals, iron levels and TfR expression (Jeong et al., 2009; Kupershmidt et al., 2009; Wang Q. et al., 2011; Golko-Perez et al., 2016; Moreau et al., 2018).

Furthermore, several studies observed an impact of SOD1 on iron metabolism. Accordingly, *in vivo* studies with SOD1.G93A mutant mice showed increased mRNA expression of ferritin, TfR1 and DMT1. These results indicate enhanced iron load, since iron is known to regulate ferritin expression (Jeong et al., 2009; Wang Q. et al., 2011). *In vitro* studies analyzing cell lysates showed an increased iron content and altered iron metabolism mediated by an impaired Akt signaling pathway. Accordingly, SOD1.G93A overexpression triggered an inactivation of Akt, activation of the transcription factor FOXO3a and subsequently increased ferritin synthesis and iron accumulation (Hadzhieva et al., 2013; Halon-Golabek et al., 2018). SOD1.G37R mutants showed *in vivo* and *in vitro* increased mRNA levels of TfR1, ferritin, DMT1 but also of mitochondrial ferritin (Jeong et al., 2009). The above-mentioned results of an increased iron content and increased TfR levels suggest an iron dyshomeostasis, which is not primarily controlled by IRE/IRP1 mechanisms (Lovejoy and Guillemin, 2014). Other studies showed diverse effects of SOD1 on the cytosolic iron sensor IRP1. SOD1 activation in G93A mutant mice showed no changes of IRP1 expression but more activated IRP1 (Jeong et al., 2009; Gajowiak et al., 2016). *In vivo*, SOD1 deficiency and the resulting oxidative stress caused IRP1 downregulation (Milczarek et al., 2017). Furthermore, in SOD1.G93A mutant mice ROS induction led to an upregulated iron import, triggering oxidative stress (Hadzhieva et al., 2013).

SOD1 is also thought to interact with the ALS-relevant protein TDP-43 (Higashi et al., 2010). Aggregates of the RNA-binding protein TDP-43 are incorporated in ubiquitinated inclusions within the neuronal cytoplasm found in ALS and in syndromes jointly named ‘neurodegeneration with brain iron accumulation’ (NBIA) (Neumann et al., 2006; Haraguchi et al., 2011). SOD1 was shown to initiate modification and accumulation of TDP-43 (Zeineddine et al., 2017; Jeon et al., 2018). In SOD1 mutant mice, iron chelators reduced TDP-43 aggregation, whereas vehicle-treated animals showed TDP-43 aggregates located in the cytoplasm of motor neurons (Wang Q. et al., 2011). Since oxidative stress-mediated accumulation of ROS fosters the TDP-43 aggregation *in vitro* (Cohen et al., 2012), iron chelator effects on TDP-43 aggregation suggest an indirect effect of iron by oxidative stress induction.

In conclusion, proteins of the iron metabolism are altered in SOD1 mutants, which partially could explain enhanced iron loading. Iron chelator effects in SOD1 models indicate an impact of iron on SOD1 pathology. Even if iron accumulation is a common feature of ALS (e.g., Moreau et al., 2018), a contribution of iron to aggregation of SOD1 or TDP-43 is yet unproven.

### Prion Protein

The prion protein (PrP) is located intracellularly and is also an important membrane-bound protein at the cell surface. Therefore, PrP is involved in exocytotic and endocytic synaptic vesicle processing as well as in signaling pathways, but also in myelination and neurogenesis (Liebert et al., 2014; Legname, 2017; Watts et al., 2018).

By using PrP knockout or PrP-overexpressing mouse models, studies showed that upregulation or downregulation of PrP levels, respectively, affect the iron homeostasis (Singh et al., 2009a,b; Pushie et al., 2011; Ashok et al., 2018). Accordingly, iron deficiency is a crucial characteristic in brains of humans, hamster and mice affected with prion pathology. Analyzing pathological brain tissue as well as scrapie-infected ScN2a and SMB cells, iron dyshomeostasis was supposedly caused by the iron sequestration in detergent-insoluble PrP-scrapie-ferritin aggregates, resulting in a decreased bio-available iron pool and a state of cellular iron deficiency. These results also explain increased amounts of total iron and Fe^2+^ as well as iron uptake proteins (Singh et al., 2009a). Another study investigated PrP-mediated iron deficiency in retinas of scrapie-injected hamsters and demonstrated an accumulation of detergent-insoluble ferritin. Furthermore, they showed a correlation of the ferritin accumulation with microglia activity. These results suggested a contribution of chronic inflammation as a side effect of PrP accumulation to functional iron deficiency (Asthana et al., 2017).

Another explanation for the influence of PrP on the iron homeostasis is that PrP operates as a plasma membrane ferrireductase. Accordingly, PrP-expressing neuroblastoma cells showed a significant increase in ferrireductase activity compared to non-transfected cells. Furthermore, mutant PrP forms showed that for an optimal ferrireductase function of PrP the presence of NADH, the copper binding octa-peptide repeat region and the linkage to the plasma membrane is needed (Singh et al., 2013). Also in HepG2-cells, expressing PrP, ferrireductase activity was indicated by an increased uptake of Fe^3+^ but not of Fe^2+^. Since Fe^3+^ uptake was significantly increased after a co-expression of PrP with metal transporter ZIP14 and DMT1, PrP is suggested to be the ferrireductase partner of both metal transporter (Tripathi et al., 2015).

So far, there is not much evidence for the influence of iron on the PrP pathology. One study using PrP-deficient HpL3-4 cells showed that Fe^3+^ but not Fe^2+^ induced accumulation of internalized PrP after iron exposure and PrP treatment of cells (Choi et al., 2013). However, it is not clarified if a direct binding to the PrP protein or indirect mechanisms triggered by iron foster the aggregation of PrP.

Vice versa, PrP was shown to influence the iron homeostasis via its ferrireductase activity and PrP-ferritin aggregates but it needs further investigation to explain if these two modes of action or indirect mechanisms such as the activation of inflammation processes lead to iron deficiency.

## Clinical Application

Iron accumulation in NDDs argues for a clinical evaluation of iron chelators as symptomatic or neuroprotective agents. Iron chelation by DFO, DFP or deferasirox (DFX) is clinically approved by the FDA for systemic conditions like acute iron intoxication and chronic iron overload^1^. DFO application is problematic due to the required continuous injection (short plasma half-life period) and dose-dependent neurotoxicity. DFX and DFP can be administered orally. DFX must be especially monitored for renal failure and DFP for agranulocytosis and neutropenia (Mobarra et al., 2016). With its ability to relocate iron and to cross the blood brain barrier, DFP presents the most promising candidate for targeting iron load in the CNS, being tested in NDDs with regional iron overload, e.g., Friedreich ataxia (e.g., Velasco-Sánchez et al., 2011; Pandolfo et al., 2014) and NBIA (e.g., Lim et al., 2018; Rohani et al., 2018).

In PD patients (*FAIR-PARK-I*, NCT00943748), DFP treatment showed benefits in motor performance and reduced MR-quantified substantia nigra iron content (Devos et al., 2014). In another trial (*DeferipronPD*, NCT01539837), administration of a lower dosage resulted in a reduced T2^∗^ MRI iron content in the dentate and caudate nucleus (Martin-Bastida et al., 2017). Based on these results, a European multicenter, parallel-group, placebo-controlled, randomized phase III trial is ongoing to assess disease-modifying effects of DFP in PD patients (*FAIRPARKII*, NCT02655315).

In a pilot trial (*SAFEFAIRALS*, NCT02164253), DFP treatment of ALS patients reduced R2^∗^ iron content in the cervical spinal cord, medulla oblongata and motor cortex. Additionally, patients showed a smaller decrease in the ALS function rating scale and in the body mass index in the first 3 months of treatment compared to the first treatment-free period (Moreau et al., 2018). Based on these results, a phase II/phase III study with a larger sample size has been initiated testing DFP (*FAIR-ALSII*, NCT03293069).

In AD, intramuscular application of DFO significantly reduced the decline of daily living skills (Crapper McLachlan et al., 1991). Besides, cognition of AD patients benefited from treatment with the metal-attenuating compound clioquinol or its derivate (Ritchie et al., 2003; Lannfelt et al., 2008). Now, AD patients are recruited for an ongoing phase II study investigating effects of DFP (*The 3D Study*, NCT03234686).

Details of the mentioned studies can be found in Supplementary Table 1. Summing up, iron chelators already showed promising results in smaller trials and this advocates for further assessment in larger studies.

## Conclusion

Current investigations focus on iron accumulation and protein aggregation as two pathological hallmarks of multiple NDDs. The interaction of both features appears to be an essential part of common neurodegeneration mechanisms. So far, iron was found to bind amyloidogenic proteins like α-synuclein, Aβ and Tau fostering their aggregation. Indirectly, iron influences disease-related proteins via oxidative stress or manipulation of their transcription and translation. On the other hand, α-synuclein, Aβ and SOD1 were found to affect iron metabolism and iron accumulation, proposing a mutual interplay and an amplification of their detrimental effects in the disease state. Even if precise effects of iron on neurodegenerative pathomechanisms remain incompletely understood, translational trials in human patients already showed beneficial effects of iron chelation as a treatment strategy for NDDs. Thus, iron may be a substantial contributor to neurodegeneration and merits further investigation as molecular and therapeutic target.

## Author Contributions

KJ developed the idea under the lead of PL, performed literature research, wrote and finalized the manuscript, and prepared the figure. A-ER and FM were involved in literature research and writing of the manuscript. PL developed the idea for this review and revised the manuscript. All authors have seen and approved the final version.

## Conflict of Interest Statement

The authors declare that the research was conducted in the absence of any commercial or financial relationships that could be construed as a potential conflict of interest.

## Footnotes

1 www.fda.gov
