# Supplementary material for: The Contribution of Iron to Protein Aggregation Disorders in the Central Nervous System
Source: Front Neurosci. 2019 Jan 22;13:15. doi: 10.3389/fnins.2019.00015 (PMC6350163; doi:10.3389/fnins.2019.00015)
Supplement: Supplementary file 1 [file Table_1.docx]

Supplementary Material

The contribution of iron to protein aggregation disorders in the Central Nervous System

Karina Joppe, Anna-Elisa Roser, Fabian Maass, Paul Lingor*

*** Correspondence:** Paul Lingor: plingor@gwdg.de

# Supplementary Material

**Supplementary Table 1: Selection of clinical trials with iron chelating agents in PD, ALS and AD.** ADAS-cog = Alzheimer's disease Assessment Scale-cognitive subscale; ADL = activities of daily living; ALSFRS = Alzheimer’s disease functional rating scale; BMI = body mass index; CSF = cerebrospinal fluid; N/A: not available; NFL = neurofilament light chain; NTB = Neuro-psychological Test Battery; UPDRS = Unified Parkinson’s disease scale.

| **Reference** | **ClinicalTrials.gov number (NCT) and study name** | **Disease** | **Iron chelator** | **Dose**  **(per day)** | **Sample size** | **Significant outcome measures** | **Study design** |
| --- | --- | --- | --- | --- | --- | --- | --- |
| Devos et al., 2014 | NCT00943748  FAIR-PARK-I | PD | deferiprone | 30 mg/kg  (p.o.) | PD n=19 (early start paradigm)  PD n=18 (delayed start paradigm) | R2* MRI  UPDRS | randomized, double-blind, placebo-controlled, parallel-group  single-center |
| Martin-Bastida et al., 2017 | NCT01539837  DeferipronPD | PD | deferiprone | 20 or 30 mg/kg  (p.o.) | PD n=22 | T2* MRI | randomized, double-blind, placebo-controlled |
| Devos and Guyon  (ongoing) | NCT02655315  FAIRPARKII | PD | deferiprone | 30 mg/kg  (p.o.) | PD n=338  (estimated) | N/A | randomized, placebo-controlled, parallel-group, European multicentre |
| Moreau et al., 2018 | NCT02164253  SAFEFAIRALS | ALS | deferiprone | 30 mg/kg/day (p.o.) | ALS n=23 | R2* MRI  ALSFRS  BMI  CSF levels of markers for NFL and oxidative stress | single-arm, pilot trial, single-center |
| Devos (ongoing) | NCT03293069  FAIR-ALS II | ALS | deferiprone | 30 mg/kg  (p.o.) | ALS n=210  (estimated) | N/A | randomized, placebo-controlled, parallel-group, multicentre |
| McLachlan et al., 1991 | N/A | AD | deferoxamine | 250 mg  (i.m.) | AD n=48 | ADL | randomized, single-blind, placebo-controlled |
| Ritchie et al., 2003 | N/A | AD | clioquinol (PBT1) | 250 mg with an increase up to  750 mg  (p.o.) | AD n=36 | ADAS-cog score (only in a subgroup analysis of the more severe stratum)  plasma Amyloid beta 1-42 levels | randomized, double-blind, placebo-controlled, parallel-group |
| Lannfelt et al., 2008 | N/A | AD | PBT2 | 50 or 250 mg  (p.o.) | AD n=78 | NTB (category fluency, trail making part B)  CSF amyloid beta 1-42 levels | randomized, double-blind, placebo-controlled |
| Soulis (ongoing) | NCT03234686  The 3D Study | AD | deferiprone | 30 mg/kg  (p.o.) | AD n=171  (estimated) | N/A | randomized, double-blind, placebo-controlled, multicentre |
